# Opuntiol Inhibits Growth and Induces Apoptosis in Human Glioblastoma Cells by Upregulating Active Caspase 3 Expression

**DOI:** 10.31557/APJCP.2021.22.11.3607

**Published:** 2021-11

**Authors:** Ambreen Ashfaque, Farina Hanif, Shabana Usman Simjee, Muhammad Furqan Bari, Shaheen Faizi, Sumbul Zehra, Talat Mirza, Sumreen Begum, Lubna Khan

**Affiliations:** 1 *Department of Physiology, Dow International Medical College, Dow University of Health Sciences, OJHA Campus, SUPARCO Road, Karachi, Pakistan. *; 2 *Department of Biochemistry, Dow International Medical College, Dow University of Health Sciences, OJHA Campus, SUPARCO Road, Karachi, Pakistan. *; 3 *H.E.J. Research Institute of Chemistry, International Center for Chemical and Biological Sciences, University of Karachi, Karachi, Pakistan. *; 4 *Department of Pathology, Dow University of Health Sciences, OJHA Campus, SUPARCO Road, Karachi, Pakistan. *; 5 *Department of Research, Ziauddin University, Karachi, Pakistan. *; 6 *Sindh Institute of Urology and Transplantation (SIUT), Pakistan. *; 7 *Institute of Biomedical Sciences, Dow University of Health Sciences, OJHA Campus, SUPARCO Road, Karachi, Pakistan. *

**Keywords:** Apoptosis, glioblastoma, opuntiol temozolomide

## Abstract

**Background:**

Glioblastoma Multiforme (GBM) is a deadly tumor with poor prognosis. Resistance to apoptosis considered as an important factor in treatment failure. Therefore, identification of new compounds that facilitates apoptosis is crucial. Natural Anti-inflammatory compounds have emerged as potential anti-cancer agents and should be explored for their apoptotic activity against GBM. Therefore, the present study aims to evaluate growth inhibitory and apoptotic activity of a natural anti-inflammatory compound “Opuntiol” against GBM cell line U87.

**Methods:**

MTT assay was performed to determine the effect of Temozolomide and Opuntiol on growth inhibition of U87 cell. While, TUNEL assay was used to assess their apoptotic activity. To further assess apoptosis, nuclear condensation and nuclear area factor (NAF) was evaluated through DAPI staining. Whereas, active caspase-3 protein expression determined using immunocytochemistry.

**Results:**

Significant growth inhibition was observed in U87 cells treated with Temozolomide (IC50 380 µM) and Opuntiol (IC_50_ 357 µM). Temozolomide (p<0.001) and Opuntiol (p<0.001) significantly improved rate of apoptosis when compared to control group. A significant decrease in NAF was also observed in Temozolomide (p < 0.05) and Opuntiol (p < 0.05) treated cells. There was a significant increase in active caspase-3 expression when observed in Temozolomide (p<0.001) and Opuntiol (p<0.05) treated groups as compared to control.

**Conclusion:**

In conclusion our findings suggests, Opuntiol repress cell viability and possess strong apoptotic activity against GBM cell line U-87. However, further mechanistic studies will be required to confirm whether it can be develop as a potential drug against GBM.

## Introduction

Glioblastoma Multiforme (GBM) is one of the most common, undifferentiated and malignant tumor of the central nervous system (Hanif et al., 2017). It accounts for about 60-70 % of all the gliomas and about 15% of primary brain tumors (Kaya-Aksoy et al., 2019). World health organization (WHO) has designated GBM as highly invasive grade IV tumor (Hanif et al., 2017).

Current standardized treatment for GBM includes chemotherapy, radiotherapy and maximal resection of the tumor which might prolong survival time to some extent, but prognosis still remains very poor. Median survival time is also approximately 14 to 15 months post diagnosis which makes it a serious public health issue (Hanif et al., 2018; Moneim et al., 2018). Therefore, there is a dire need to identify new therapeutic compounds or drugs which can target the disease and improve the prognosis. 

GBM is a result of many deregulated signaling pathway which occurs due to several genetic alterations, epigenetic modifications, point mutations, deletions, or amplification (Alpade et al., 2017). Following major possible events that are found to be liable to stimulate gliomagenesis includes, Over expression of growth factors, Angiogenesis, Loss of cell cycle control, Genetic instability, Invasion and metastasis, Evasion from apoptosis (Shi et al., 2017; Nakada et al., 2011). As the defects of apoptosis are not only a common occurrence in oncogenesis but also contribute to poor drug response therefore they have been considered as important therapeutic target (Hanif et al., 2014) and also the main target of the present study. 

Studies suggest natural compounds might prove to become a good source to develop and discover new antineoplastic agents (Liu et al., 2015). Plant derived molecules have played an important role in inducing apoptosis in cancer cells and cell cycle arrest thus can play a key role in controlling progression and prevention of cancer (Prasad et al., 2018). Natural products have also got much attention for cancer chemotherapy due to their high effectiveness and low toxicity. 

Opuntiol is a naturally occurring compound, isolated from the plant of opuntia dellinii haw (Siddiqui et al., 2016). It possess several medicinal properties like gastric ulcer treatment, gonorrhea, intestinal spasm, diabetes, asthma, whooping cough, and many other diseases (Siddiqui et al., 2016; Roome et al., 2019). It has been reported that this compound possesses strong anti-inflammatory properties and can be of potential use in the management of inflammation (Roome et al., 2019).

It is well established that carcinogenesis is multi-step process which involves sequential events of initiation, promotion then progression, in which inflammation acts like a promoter and causes accumulation of more mutations via preventing apoptosis and facilitate proliferation of these mutant cells thus providing pre-neoplastic and neoplastic cells growth advantages by Darwinian selection. Epidemiological evidence have also shown that persistent inflammatory stimuli are commonly linked with an increased risk of cancer development (Philip et al., 2004).Thus this study aims to testify the potential effect of Opuntiol an anti-inflammatory compound against cancer specifically glioblastoma.

## Materials and Methods

*Preparation of Test Compound*

Test compound Opuntiol was obtained from our chemist collaborators at International Center for chemical and Biological Sciences. Opuntiol was isolated from the methanolic extract of plant opuntia dellini cladode as previously reported by our collaborators (Siddiqui et al., 2016). Standard drug Temozolomide was purchased from Sigma Aldrich. Temozolomide and Opuntiol were dissolved in Dimethyl Sulfoxide (DMSO) to prepare stock-solution of 25 mM. The stock was stored at -20^o^C until further use for making working dilutions. The final concentration of DMSO used was less than 0.1 %.

*Cell Culture*

Human Glioblastoma cell line U-87MG or U87 was purchased from American type tissue culture collection (ATCC). Briefly, Cells were cultured in 25 cm^2^ flasks containing complete media (Dulbecco’s Modified Eagle Medium (DMEM) + fetal bovine serum (FBS) + Penicillin and streptomycin) and were then transferred to a humidified-atmosphere at 37^o^C and 5% of CO_2_.

*3-(4,-5-Dimethyl-Thiazol-2-Yl)-2,5-Diphenyl-Tetrazolium Bromide (MTT) Assay*

MTT assay was exploited to analyze the effect of Opuntiol and Temozolomide on growth inhibition (%) of the cells (Hanif et al., 2013). Cells were cultured in 96 well plates, after time period of 24 hours, when monolayer was formed, cells were treated with different test concentrations of Temozolomide (25µM, 50µM, 100µM, 200µM and 400µM) and Opuntiol (25µM, 50µM, 100µM, 200µM and 400µM) followed by incubation of 48 hours at temperature of 37^o^C in 5 % CO_2_ incubator. Cells treated with DMSO served as vehicle control. Then 100 µL of MTT (0.5 mg/mL) was added to the wells and cells were further incubated for 3 hours. After incubation, supernatant was removed and 100 µL of DMSO was added to cells and absorbance was recorded spectrophotometrically at 490 nm of wavelength on Elisa Reader (BioTek U.S). The assay was done in triplicates at least thrice. The percentage growth inhibition was calculated using the following formula, 

Percentage growth inhibition of cells= 100-{(At - Ab)/(Ac - Ab)}x100

IC_50_ was calculated using the following formula:

IC_50_ = (X2 - X1) X (50 - Y1) / (Y2 - Y1) + X1

Where, X1 and X2 represents higher and lower concentrations that were used. Y1 and Y2 represents mean % of live cells at the higher and lower-used concentrations (Mathieu et al., 2008).

Analysis of Gross Morphological Features of the U87 Cells Following Treatment with Opuntiol and Temozolomide:

Cells were grown in 25 cm^3^ flasks and treated with vehicle control and IC_50_-concentration of Opuntiol and Temozolomide for 48 hours. The images were captured at 20 X magnifications under inverted microscope using phase contrast microscopy.

*Nuclear-Area Factor for Apoptosis Assessment*

Nuclear area factor (NAF) was also measured to assess apoptosis, Cells were grown on the cover slips and exposed with the IC50 dose of Temozolomide and Opuntiol. After 48 hours of exposure, media was aspirated, cells were fixed in 2% paraformaldehyde (PFA). Post fixation, cells were exposed to 4′,6-diamidino-2-phenylindole (DAPI) stained with NucBlue^®^ Fixed Cell ReadyProbes® Reagent (ThermoScientific, USA). Five Images were taken from random areas employing fluorescence microscope at 40X magnification. Images were processed using ImageJ software. NAF was calculated with:

Following formula:

Nuclear area factor = circularity × object area (DeCoster et al., 2007).

Terminal Deoxynucleotidyl Transferase dUTP Nick End Labeling (TUNEL) Assay for Apoptosis: 

The terminal dUTP nick-end-labeling (TUNEL) assay kit (Promega Corporation, USA) was used to detect DNA fragmentation, a hallmark of apoptosis in treated and control U87 glioblastoma cells as per manufacturer’s protocol. Cells with dark brown stained nuclei considered apoptotic. Quantification of apoptotic cells was done by counting total-cells and apoptotic cells under the microscope in five randomly selected fields (Yang et al., 2002). Data is expressed as means ± SEM of three different experiments. 

*Immunocytochemistry for Analyzing Active Caspase-3 Expression in U87 Cells*

For immuno-cytochemical analysis cells were plated on chamber slides and were treated with Opuntiol, Temozolomide and DMSO (vehicle control) for 48 hours at 37^o^C and 5% CO_2_. Following 48 hours treatment cells were fixed with PFA (4%), washed with Phosphate-buffered saline (PBS) and blocked with blocking solution (ROTI®Block) at 37ºC for 1 hour. Next, cells were washed with PBS again and incubated with rabbit-anti-cleaved-caspase-3 antibody (Invitrogen) at 4^o^C overnight. Next day, cells were washed with PBS and then incubated for 1 hour at room temperature with secondary antibody Alexa-Flour^®^546 anti-rabbit IgG (Invitrogen) in PBS. Cells were then washed again and counterstained with DAPI. The stained slides were then mounted with mounting media and viewed under a fluorescent-microscope. Quantification studies of the images were done using ImageJ-software (National Institutes of Health, USA) (Collins et al., 2007).

*Statistical Analysis*

All the experiments were performed thrice in triplicates. The results obtained from experiments were analyzed with SPSS-21 software. Data is expressed as mean ± standard-error of the mean of separate experiments (n ≥3) and compared by one-way analysis of variance (ANOVA) and followed by Bonferoni post-hoc test. P <0.05 considered statistically-significant.

## Results

*Growth inhibitory effect of Temozolomide and Opuntiol on U87 cells*

For assessing growth inhibitory effect of Opuntiol and Temozolomide, MTT assay was used. 

Aforementioned doses in the methodology section were used to treat U87 cells for 48 hours and obtained data was analyzed with SPSS, using one way ANOVA and post-hoc (bonferoni) test for the multiple comparisons.

**Figure 1:**
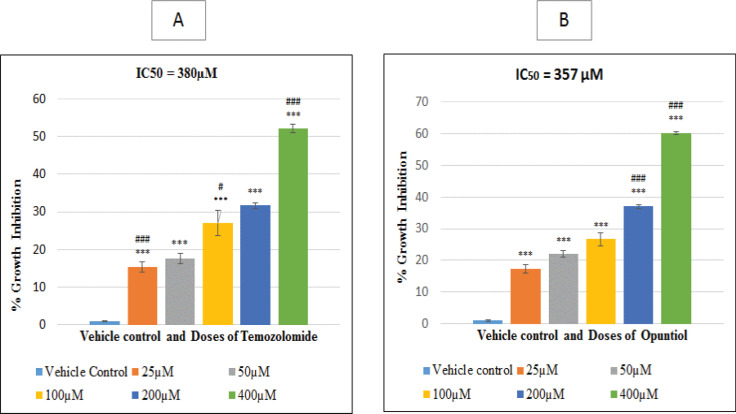
Effect of Temozolomide (A) and Opuntiol (B) on Growth Inhibition of U87 Cells. Bars in above graph represents the mean ± S.E.M of 3 independent experiments. Cells were treated with the vehicle control and different doses of Temozolomide and Opuntiol for 48 hours followed by MTT assay. Growth inhibition was observed for both Temozolomide and Opuntiol in dose dependent manner and IC50 was achieved at 380uM and 357uM respectively. Significant differences was observed in treated group as compared to control groups, which is indicated by ***P <0.001, ** p < 0.01 and *p < 0.05. Further bonferoni’s post hoc test was applied which revealed a significant difference within different consecutive groups indicated with ###p<0.001 and #p<0.05 among various doses of Temozolomide and Opuntiol

It was observed that standard drug Temozolomide significantly inhibited growth of U87 cells in dose dependent manner as compared to vehicle control cells (P<0.001). The IC_50_ value calculated for Temozolomide was 380µM. The posthoc test showed a significant difference towards growth inhibition among Temozolomide 200µM and 400µM (P ≤ 0.05). While no significant difference was observed among, Temozolomide 25µM and 50µM, and Temozolomide 100µM and 200µM (P > 0.05) (Figure 1A).

Opuntiol also significantly inhibited growth of U87 cells in dose dependent manner as compared to vehicle control cells (P<0.001). The IC_50_ value calculated was 357µM. The posthoc bonferoni test was applied to analyze difference between the Opuntiol treated groups which showed a significant difference in growth inhibition among Opuntiol 100 µM and 200µM (P ≤ 0.05) and Opuntiol 200µM and 400µM (P ≤ 0.05) While no significant difference was observed among Opuntiol 25 µM and 50 µM (P > 0.05) (Figure 1B).

*Morphological evaluation of U87 cells treated with Temozolomide and Opuntiol*

**Figure 2:**
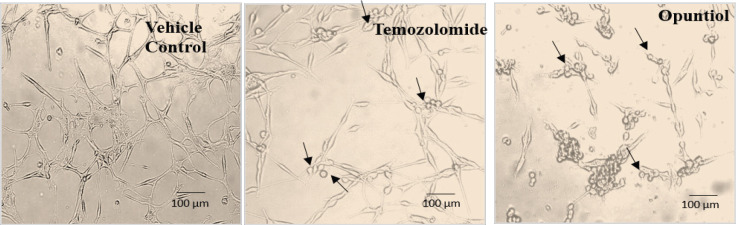
Photomicrographs Representing the Effect of Drug and Compounds on the Morphological Changes in U87Cells. The human U87 cells were treated with Temozolomide and Opuntiol for 48 hours, the cells were then visualized by phase contrast microscopy. Photomicrographs showing apoptotic changes in cellular morphology of drug treated cells, where they have lost their connections and become rounded; indicated with arrowheads. All experiments were done in triplicate and thrice

U87 human glioblastoma cells were treated with DMSO (Vehicle control) and IC_50 _doses of Opuntiol and Temozolomide for 48 hours. Then cells were visualized at 20x magnification under phase contrast microscopy for assessment of cell morphology. Morphological changes indicative of apoptosis were detected among cells treated with Opuntiol and Temozolomide at 48 hours exposure. Predominantly cells became rounded from star shape, loosed their connections, loosed their processes, shrinked in size, loose their adherence from walls and neighboring cells and detached from surfaces (Figure 2).

*Evaluation of apoptosis via TUNEL ASSAY*

**Figure 3:**
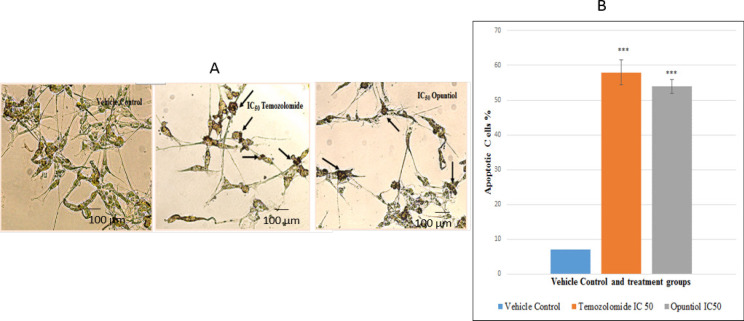
Photomicrographs and Graphical Illustration Showing Increased Apoptosis of U87 Cells via TUNEL Assay. (A) The human U87 cells were exposed to IC50 doses of Temozolomide and Opuntiol for 48 hours and observed under inverted microscope. TUNEL assay was applied to detect apoptotic cells. Dark brown stained cells are symbolic of apoptosis in above images which are indicated with arrow heads. Experiments were performed thrice in triplicates. (B) Fig B shows graphical representation of TUNEL assay showing mean ± S.E.M of 3 independent experiments and percentages of apoptotic cells. Apoptosis was significantly increased in Temozolomide and Opuntiol treatment groups as compared to the control group indicated by ***p< 0.001

Cells were treated with vehicle control and IC_50 _doses of Temozolomide and Opuntiol for 48 hours. Further TUNEL assay was applied to evaluate the cells for induction of apoptosis and photomicrographs were captured under inverted microscope at 20x magnification. Significant increase was observed in number of apoptotic cells in treatment groups i.e. Temozolomide (p<0.001) and Opuntiol (p<0.001) as compared to control group. Cells treated with Temozolomide and Opuntiol showed dark brown stained nuclei indicating nicks in Deoxyribonucleic acid (DNA) and thus apoptosis which was not observed in the control group. Around 58% ± 3.5% of cells were apoptotic in Temozolomide group as compared to control group where only around 7% ± 1% cells were seen apoptotic. Whereas in treatment group of Opuntiol around 54% ± 2%cells were apoptotic (Figure 3 A and B).

*Nuclear Area Factor (NAF)*

**Figure 4:**
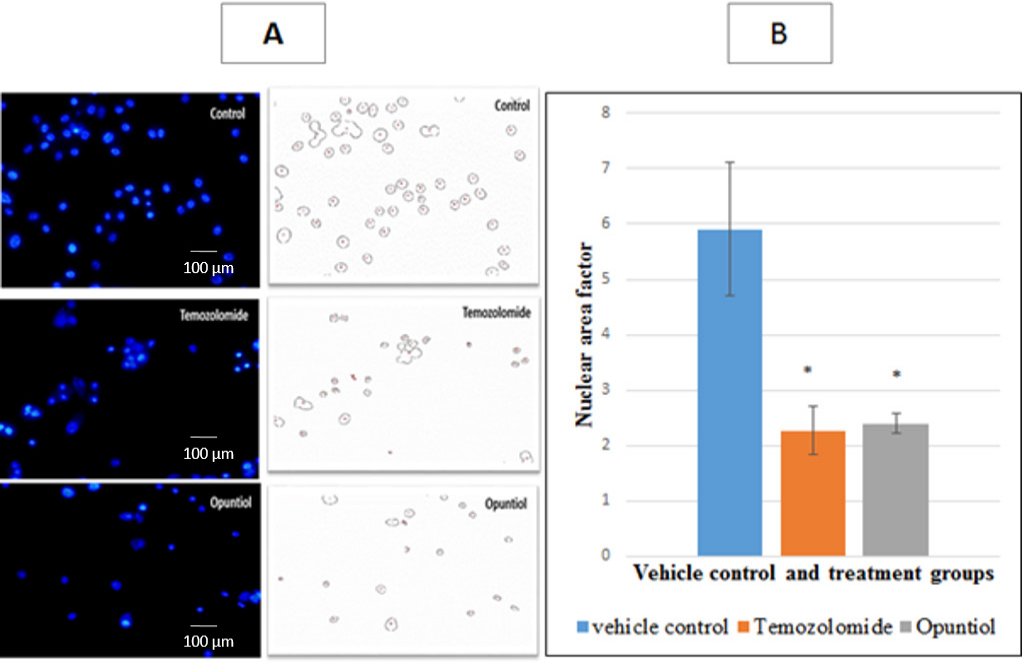
Photomicrographs and Graphical Illustration Showing Nuclear Area Factor in Treatment and Control Group. (A) U87 human glioblastoma cells were treated with IC50 doses of Temozolomide and Opuntiol for 48 hours. Nuclear area factor was calculated in treatment exposed cells and were compared to control group. Apparent increase was observed in nuclear condensation thus decreased NAF in Temozolomide and Opuntiol treatment groups as compared to vehicle control. (B) Fig B shows Graphical representation of mean nuclear area factor with ± S.E.M of 3 independent experiments. Nuclear area factor was significantly decreased in cells treated with Temozolomide and Opuntiol as compared to control group (p<0.05).

DAPI nuclei staining was used to analyze nuclear area factor as nuclear condensation is one of the notable features of apoptosis. After 48 hours of treatment a dramatic decrease was observed visually in NAF in Temozolomide and Opuntiol treatment groups as compared to the control group. Condensation was further confirmed by image J analysis, which revealed significant decrease in NAF from 5.9 ± 0.44 in vehicle control to 2.2 ± 1.2 in Temozolomide and 2.4 ± 0.17 in Opuntiol treated cells (P < 0.05) (Figures 4 A and B).

*Active Caspase-3 Expression after Treating U87 Cells with Temozolomide and Opuntiol*

**Figure 5:**
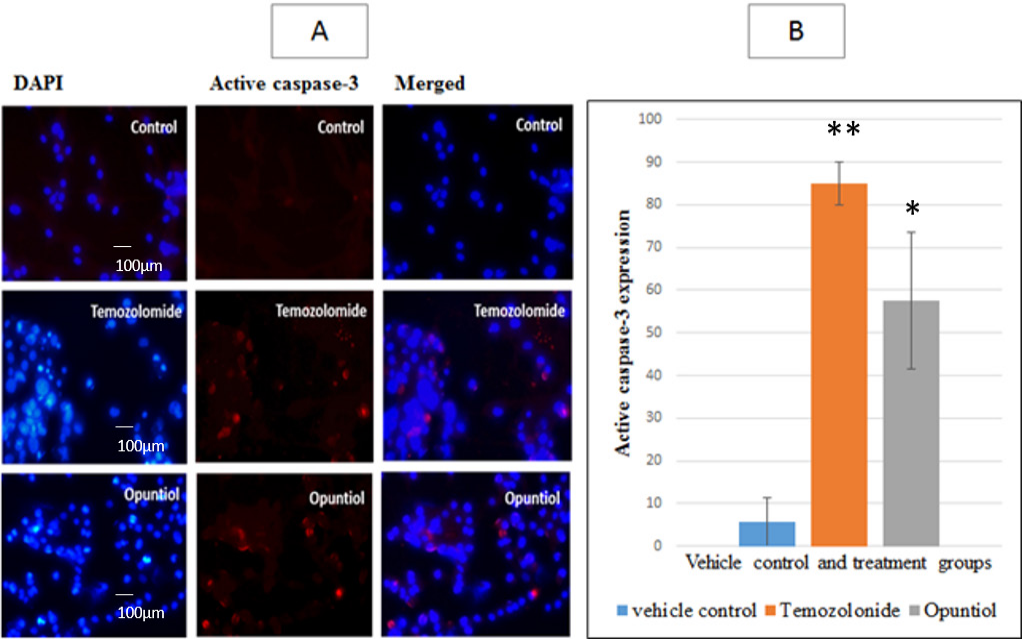
Photomicrographs and Graphical Illustration Representing the Intensity of Expression of Active Caspase-3 Protein in U87Cells. (A)The human U87 cells were treated with IC50 doses Temozolomide and Opuntiol for 48 hours, the cells were then visualized by fluorescence microscopy. Where the images on the right showing fluorescence intensity (red) plotted with cell nucleus (blue). Photomicrographs showing noticeable expression of active caspase-3 in treated groups, indicated with arrowheads. All experiments were done thrice in triplicate. (B) Graphical representation of active caspase-3 expression followed by 48 hours treatment with Temozolomide and Opuntiol, showing mean ± S.E.M of 3 independent experiments. Active caspase-3 expression was significantly increased in Temozolomide and Opuntiol treatment groups as compared to control group, indicated by ** p < 0.01 and *p < 0.05

Active caspase-3 expression was analyzed after 48 hours of cells treatment with IC50 doses of Temozolomide and Opuntiol via immunoflourescence. Photomicrographs analysis through image J revealed significant (p<0.001) increase in active caspase-3 expression in Temozolomide group (88 ± 5.1%) as compare to vehicle control group (5.6 ± 5.6). Expression was further observed in Opuntiol treated group where it also showed significant upturn of active caspase-3 expression (p<0.05), where the integrated density of expression was 57.6 ± 16 (Figures 5 A and B).

## Discussion

In spite of advance treatment approaches and research in the field of oncology, glioblastoma still remains a deadly tumor with 14-15 months survival rate due to its aggressive nature and recurrence properties. Over the years investigations has been done to find out bioactive molecules which can halt the progression of tumor and improve the prognosis but unfortunately still with only limited success (Hanif et al., 2018). Role of dysregulated apoptotic cell machinery is well established in cancer progression and therefore is an attractive target in cancer therapy (Koff et al., 2015).

The present study demonstrates significant apoptotic activity of a natural anti-inflammatory compound Opuntiol against glioblastoma cells. Most of the previous studies have reported significant role of Opuntiol in inflammation and other diseases but none of the study has shown its importance towards its role in apoptosis more specifically against glioblastoma cells. 

It has been confirmed by previous studies that inflammation is closely related to oncogenesis (Kim et al., 2011). Therefore, we hypothesized that our test compound “Opuntiol” which possess strong anti-inflammatory activity might also have anti-cancer activities. The test compound (Opuntiol) of the present study was isolated from the plant of opuntia dellinii (Siddiqui et al., 2016). It has been reported recently that other species of the plant cactus from which opuntiol has also been extracted also possess anti-inflammatory as well apoptotic properties against oral carcinoma cells (El-Mostafa et al., 2014; Veeramani et al., 2019).

Results of present study revealed that Temozolomide and Opuntiol induces growth inhibition of U87 cells in dose dependent manner as compared to vehicle control. Our results are in accordance with the previous study that reported growth inhibition activities of anti-inflammatory drugs and compounds either synthetic or natural origin against different cancer cells (Aziz et al., 2019; Naselli et al 2014). Non-steroidal anti-inflammatory drugs believe to have role in inducing apoptosis in several cancer cell lines and also has role in reducing the viability of GBM cells (Hanif et al., 2014). It has also been reported that naturally occurring anti-inflammatory compounds also possess anti- proliferative, apoptotic and anti-cancer activity (Naselli et al 2014). To further confirm whether Opuntiol mediated growth inhibition involves apoptosis or it is just a cytotoxic effect, we employed multiple assays to confirm apoptotic death of U87 cells.

 Morphology of U87 cells was observed after treatment with Temozolomide and Opuntiol, morphological changes of U87 cells were aligning with all the apoptotic changes as described previously (Hanif et al 2018).

Briefly, U87 possesses distinct morphological features of forming processes, making contact with neighboring cell and have star shape morphology, while under apoptotic condition it was observed that cells have lost their adherence and contact with neighboring cell and became rounded in appearance (Hanif et al., 2018). DNA fragmentation which is another important hallmarks of apoptosis was also evaluated using terminal dUTP nick-end-labeling (TUNEL) assay kit and it further confirmed the presence of apoptosis in treated cells. The assay detects apoptotic DNA fragmentation by identifying double-stranded DNA breaks and labels them with biotinylated nucleotides at 3’ OH end by recombinant enzyme rTdT and results in dark brown nuclei which are indicative of apoptosis and can be visualized under phase contrast microscopy (Hanif et al., 2014; Hanif et al., 2018). In the present study, U87 cells treated with Temozolomide and Opuntiol showed dark brown stained nuclei, indicating nicks in DNA and thus apoptosis. The results are in accord with previous study where ant-inflammatory compound and Temozolomide induced DNA fragmentation of U87 cells which was not observed in the control group, the results were also statistically significant on analysis (Hanif et al., 2018). A previous study also supports our results, they have reported apoptotic activity of extracts of opuntia ficus indica L against cancer cell lines using TUNEL assay. The plant belongs to the same family from which our test compound Opuntiol has been extracted and also possess anti-inflammatory activity (Becer et al., 2018).

DAPI nuclei fluorescent staining was also used to analyze nuclear area factor, as nuclear condensation is one of the notable features of apoptosis and it is an early indicator of changes in the cell morphology during apoptosis (DeCoster et al., 2007). Prominent decrease was observed in NAF in Temozolomide and Opuntiol treated groups as compared to control group. It has been reported previously that increase in NAF is associated with Temozolomide resistance, therefore, the decrease in NAF that we have observed may further confirms the significant apoptotic activity and sensitivity of U87 with Temozolomide and opuntiol (Tiek et al., 2018).

Lastly, the final confirmation of apoptosis by our test compound was achieved by performing caspase-3 protein expression which is an ultimate executioner caspase and an important molecular marker of apoptotic pathways either intrinsic or extrinsic (Hanif et al., 2014). Treatment with both Temozolomide and Opuntiol showed significant upturn of active caspase-3 expression in U87 cells under influence of Temozolomide and Opuntiol in treatment groups. Our result is supported by previous study in which anti-inflammatory drugs and Temozolomide induce apoptosis by caspase-3 activation in gastric cancer cell line as well as in U87 GBM cells (Hanif et al., 2014; Naselli et al 2014). In conclusion our findings suggests Opuntiol represses cell viability and possess strong apoptotic activity against GBM cell line U87. However, to completely understand its mechanism of action detailed studies at the molecular levels are required. The results should also be validated in in vivo GBM model. 

## Author Contribution Statement

The authors confirm contribution to the paper as follows: Study conception and design: FH; Experimental work: AM, SZ, SB, LK, FB; Data analysis: AM, FH, SUS, TM; Chemical compound preparation: SF; draft manuscript preparation: AM, FH. All authors reviewed the results and approved the final version of the manuscript. 

## References

[B1] Aldape K, Zadeh G, Mansouri S, Reifenberger G, von Deimling A (2015). Glioblastoma: pathology, molecular mechanisms and markers. Acta Neuropathol.

[B2] Aziz A, Hanif F, Majeed S, Iftikhar K, Simjee SU (2019). N-(2-hydroxyphenyl) acetamide (NA-2) elicits potent antitumor effect against human breast cancer cell line (MCF-7). Toxicol In Vitro.

[B3] Becer E, Kabadayı H, Meriçli F (2018). Apoptotic effects of Opuntia ficus indica L Seed Oils on Colon Adenocarcinoma Cell Lines. Proceedings.

[B4] Collins T (2007). Image J for Microscopy. BioTechniques.

[B5] DeCoster MA (2007). The nuclear area factor (NAF): a measure for cell apoptosis using microscopy and image analysis. Modern Res Edu Topics Microscopy.

[B6] El-Mostafa K, El Kharrassi Y, Badreddine A (2014). Nopal cactus (Opuntia ficus-indica) as a source of bioactive compounds for nutrition, health and disease. Molecules.

[B7] Hanif F, Perveen K, Jawed H, Simjee Su (2013). In vitro growth inhibition Of U87 glioblastoma cells by N-(2-Hydroxy Phenyl) acetamide. Int J Med Pharm.

[B8] Hanif F, Perveen K, Jawed H (2014). N-(2-hydroxyphenyl) acetamide (NA-2) and Temozolomide synergistically induce apoptosis in human glioblastoma cell line U87. Cancer Cell Int.

[B9] Hanif F, Muzaffar K, Perveen K, Malhi SM, Simjee SU (2017). Glioblastoma multiforme: A review of its epidemiology and pathogenesis through clinical presentation and treatment. Asian Pac J Cancer Prev.

[B10] Hanif F, Perveen K, Malhi SM, Jawed H, Simjee SU (2018). Verapamil potentiates anti-glioblastoma efficacy of temozolomide by modulating apoptotic signaling. Toxicol In Vitro.

[B11] Kaya-Aksoy E, Cingoz A, Senbabaoglu F (2019). The pro-apoptotic Bcl-2 family member Harakiri (HRK) induces cell death in glioblastoma multiforme. Cell Death Discov.

[B12] Kim BH, Kim CI, Chang HS (2011). Cyclooxygenase-2 overexpression in chronic inflammation associated with benign prostatic hyperplasia: is it related to apoptosis and angiogenesis of prostate cancer?. Korean J Urol.

[B13] Koff JL, Ramachandiran S, Bernal-Mizrachi L (2015). A time to kill, targeting apoptosis in cancer. Int J Mol Sci.

[B14] Liu Y, Bi T, Wang G (2015). Lupeol inhibits proliferation and induces apoptosis of human pancreatic cancer PCNA-1 cells through AKT/ERK pathways. Naunyn-Schmiedeberg’s Arch Pharmacol.

[B15] Mathieu V, De Nève N, Le Mercier M (2008). Combining bevacizumab with temozolomide increases the antitumor efficacy of temozolomide in a human glioblastoma orthotopic xenograft model. Neoplasia.

[B16] Moneim RA, Abdel-Rafei M, Hassan H, El-Zawahry I, Hashem WB (2018). Prognostic significance of MGMT promoter methylation in Egyptian GBM patients: A Single-institution Experience. Asian Pac J Cancer Biol.

[B17] Nakada M, Kita D, Watanabe T (2011). Aberrant signaling pathways in glioma. Cancers.

[B18] Naselli F, Tesoriere L, Caradonna F (2014). Anti-proliferative and pro-apoptotic activity of whole extract and isolated indicaxanthin from Opuntia ficus-indica associated with re-activation of the onco-suppressor p16INK4a gene in human colorectal carcinoma (Caco-2) cells. Biochem Biophys Res Commun.

[B19] Philip M, Rowley DA, Schreiber H (2004). Inflammation as a tumor promoter in cancer induction. Semin Cancer Biol.

[B20] Prasad N, Sabarwal A, Yadav UC, Singh RP (2018). Lupeol induces S-phase arrest and mitochondria-mediated apoptosis in cervical cancer cells. J Biosci.

[B21] Roome T, Aziz S, Razzak A (2019). Opuntioside, opuntiol and its metallic nanoparticles attenuate adjuvant-induced arthritis: novel suppressors of toll-like receptors-2 and-4. Biomed Pharmacother.

[B22] Shi J, Dong B, Cao J (2017). Long non-coding RNA in glioma: signaling pathways. Oncotarget.

[B23] Siddiqui F, Abidi L, Poh CF (2016). Analgesic potential of Opuntia dillenii and its compounds Opuntiol and Opuntioside against pain models in Mice. Rec Nat Prod.

[B24] Siddiqui F, Naqvi S, Abidi L (2016). Opuntia dillenii cladode: Opuntiol and opuntioside attenuated cytokines and eicosanoids mediated inflammation. J Ethnopharmacol.

[B25] Sreekanth D, Arunasree M, Roy KR (2007). Betanin a betacyanin pigment purified from fruits of Opuntia ficus-indica induces apoptosis in human chronic myeloid leukemia Cell line-K562. Phytomedicine.

[B26] Tiek DM, Rone JD, Graham GT (2018). Alterations in cell motility, proliferation, and metabolism in novel models of acquired temozolomide resistant glioblastoma. Sci Rep.

[B27] Veeramani kandan P, Dhineshkumar E, Karthikeyan R (2019). Isolation and characterization of opuntiol from Opuntia Ficus indica (L Mill) and its antiproliferative effect in KB oral carcinoma cells. Nat Prod Res.

[B28] Yang H, Bhat GK, Sridaran R (2002). Clinostat rotation induces apoptosis in luteal cells of the pregnant rat. Biol Reprod.

